# Framing effects in factors influencing health professional staff decisions to leave or stay working in the UK National Health Service

**DOI:** 10.1108/JHOM-10-2025-0638

**Published:** 2026-02-20

**Authors:** Andrew Keith Weyman, Rachel O'Hara, Richard Glendinning, Deborah Roy, Joanne E. Coster

**Affiliations:** Department of Psychology, University of Bath, Bath, UK; School of Health and Related Research, The University of Sheffield, Sheffield, UK; University of Bath, Bath, UK

**Keywords:** Framing effects, UK healthcare staff retention, Reasons to leave, Reasons to stay, Paired comparisons

## Abstract

**Purpose:**

In common with other health care providers internationally, health professional retention represents a key challenge for the UK National Health Service (NHS). This study set out to quantify secondary care health professionals’ ratings of the relative importance of widely cited influences on staff retention. The method of paired comparisons was used to determine whether ratings vary between the principal job families (doctors, allied health professionals, nurses/midwives, paramedics and nursing support) and to test whether reasons for leaving fully explain what needs to change to retain staff.

**Design/methodology/approach:**

Independent samples of NHS staff (*N* = 1958; *N* = 1994) completed one of two paired comparisons tasks, framed as reasons why staff in their job-family leave or what needs to change to encourage staff in their job-family to stay, referenced to eight variables: working hours, pay, workload, staffing levels, mental health/stress, work–home life balance, time pressure and recognition of contribution.

**Findings:**

Across all job-families and both frames, staffing levels and mental health/stress emerged as top priorities. However, priorities showed variability by profession and framing.

**Practical implications:**

A comprehensive perspective on retention requires consideration of both why staff leave and what needs to change to encourage them to stay. Evidence of job-family differences in priorities for attention suggests the need for a segmented approach to intervention.

**Originality/value:**

This study is believed to be the first large sample comparison of secondary care job family perspectives on why staff leave the NHS and what needs to change to encourage them to stay.

## Introduction

The issue of staff shortages within the UK National Health Service (NHS) has become more prominent in the post COVID-19 pandemic period. High rates of staff turnover and, most saliently, high rates of early and late career exits from NHS employment are not a new feature (Shields and Ward, 2001; Finlayson *et al.*, 2002; Bamford and Hall, 2007). However, the unprecedented demand for care during the pandemic and subsequent spiralling treatment waiting lists has increased interest in health professionals’ well-being, in particular, their capacity and resolve to remain in NHS employment (Holmes, 2022; O'Dowd, 2021; Oliver, 2022). While NHS vacancy statistics show a small reduction in the overall number of vacancies from a peak of 130,000 in December 2022 to 112,000 in June 2023 (NHS Employers, 2023), vacancy rates amongst key groups remain high, in particular nurses (Deveraux, 2023). For NHS employers, retention “… remains a key issue as leaver rates remain historically high and the NHS continues to lose experienced long-serving staff.” (NHS Employers, 2023). Staff retention represents an organisational risk management issue with regard to the sufficiency of staff resource to meet the increasing demand and deliver safe care, but also with respect to a duty of care to protect the health and well-being of employees (Health and Safety at Work etc Act, 1974). Effective risk mitigation in each regard relies on gathering good quality evidence on variables that influence staff disposition to leave or stay.

Previous research has identified a range of variables driving early exit from the NHS, including workload, mental health/stress, satisfaction with pay, work-life balance and working hours (Weyman *et al.*, 2020). However, studies of single professions and of small, modest sample size predominate. While these studies afford some insight and exhibit a degree of alignment in variables identified, direct comparison of different job families is limited and there is little scope for pre and post COVID-19 pandemic comparison. Moreover, limited evidence on the distinction between why staff leave and what might need to change to motivate/support staff to stay promotes the implicit assumption that they are synonymous. A number of studies within the health sector and beyond have questioned the notion that reasons to leave are a mirror image of reasons to stay (Coombs *et al.*, 2010; Cho *et al.*, 2009). For example, a study of allied health professional returnees reported modest alignment between their reasons for leaving (workload, pay, lack of flexible hours, caring responsibilities) and their reasons for returning (availability of flexible hours, career progression, skill development and pension) to NHS employment (Coombs *et al.*, 2010). In order to determine priorities for intervention, it is important to establish whether reasons for leaving can fully explain what needs to change in order to retain staff.

Although a number of studies identify negative experiences of working through the COVID-19 pandemic as driving intentions to leave across professions and healthcare organisations (Dunning *et al.*, 2024; Kennedy *et al.*, 2022; Falatah, 2021), most studies have involved single healthcare professions or roles. There is a need for larger scale research examining the influences on retention since the emergence of COVID-19 that will also support a better understanding of whether the profile of key variables presents as consistent or different across the different health professional job families.

There is considerable evidence regarding the role of workload and burnout on turnover in nursing (Chang *et al.*, 2025; Buckley *et al.*, 2025), including intention to leave the profession (Bouranta and Kafetzopoulos, 2025) and variation between different international healthcare systems (Bruyneel *et al.*, 2025). Reviews of studies examining intention to leave (profession) and attrition for a range of allied health professions internationally (Roth *et al.*, 2024; Yeoh *et al.*, 2024) identified working hours, workload, support and burnout as factors influencing stay/leave intentions. Adequate pay was identified as an essential consideration to stay, but it was not the only consideration (Roth *et al.*, 2024). A review of studies exploring the determinants of turnover intentions among doctors highlighted stress and burnout as contributors to increased turnover (Wang *et al.*, 2025). However, the authors concluded that there is a significant gap in the literature addressing doctor turnover compared to broader healthcare workers. Seathu Raman *et al.* (2024) identified dissatisfaction with working hours, shift work patterns and administrative demands as key factors contributing to turnover amongst hospital doctors. They also note a gap in the literature regarding the consideration of retention and the reasons why doctors remain. Studies exploring reasons for paramedic attrition identify organisational issues such as leadership, pay, excessive working hours and limited access to flexible working among the most frequently reported reasons for leaving the profession (Middleton *et al.*, 2025).

Findings across the various evidence reviews appear in broad agreement regarding the key factors influencing stay/leave decisions for health professionals. However, a consistent feature across the evidence for all health professionals is the lack of differentiation between reasons to leave and reasons to stay that could inform strategies for retention. There is also considerable definitional heterogeneity across the constituent studies that makes it difficult to identify the relative importance of specific factors for the different health professions.

## Research methodology

The aim of the research presented in this paper is to identify the relative importance and alignment of reasons why staff leave the NHS and what needs to change to encourage staff to stay. Also, to determine the degree of consistency across different health professions. The method of paired comparisons was used to test whether reasons for leaving can fully explain what needs to change in order to retain staff and to determine whether ratings vary between the principal job families (doctors, allied health professionals, nursing professionals (including midwives), paramedics and nurse non-professionals (support staff)).

### Study design

The study is a quantitative, cross-sectional, observational survey using Thurston's Case V paired comparisons scaling method (Thurstone, 1927). The approach mirrors previous research into NHS staff retention (Weyman *et al.*, 2020, 2023) and was purposely conceived to determine the relative salience of different variables and proportionately how much more or less important one variable is than another (Thurstone, 1927; Bock and Jones, 1968).

Paired comparisons are particularly well suited for scaling entities for which objective values are unknown or unknowable, this being the case for variables impacting on staff leave and stay behaviour. Its principal advantages over widely applied alternatives are (1) a weighted scale affords greater insight than simple ordinal ranking, (2) it is less prone to produce halo and horn effects than Likert scales, (3) it imposes a lower cognitive load and is less time consuming to perform than alternative sorting techniques, e.g. Q-Method and Repertory Grid, (4) it allows statistical testing of within-participant reproducibility reliability and (5) the reproducibility reliability of the output (Bock and Jones, 1968; Comer *et al.*, 1984). It has been widely applied in a range of domains, including determining priorities for healthcare resource allocation (Skedgel and Regier, 2015; Skedgel *et al*., 2015a, b; Gijsbers *et al*., 2021; Ryan *et al.*, 2001).

The technique involves independently presenting each participant with randomised pairs of variables from a defined set. This is done for all permutations of pairings; a set of eight variables requires 28 separate comparisons. For each pairing, participants select the variable judged to be of greater importance with reference to a defined criterion. Summation of the frequency with which each variable is rated as having greater importance than all of the other variables within the item set forms the basis of a scaled output for each participant. Where concordance (agreement) between participants is strong, this allows the generation of a scale that can be considered to characterise the group’s ratings. This allows between-group comparison of variable rankings and relative weightings, e.g. doctors compared with nurses. Standard statistical tests are used to determine within-participant consistency, within-group concordance and between-group concordance (Thurstone, 1927; Sjoberg, 1967).

1. Determination of the variable set

The variable set comprised eight variables: *Staffing levels*, *working hours*, *mental health/stress*, *pay*, *time pressure* and *recognition of contribution*, selected on the basis of their primacy within published findings on health professional retention (Weyman *et al.*, 2020; see Appendix Table A1). Following consultation with the project advisory panel (which included stakeholders from NHS England/Improvement; health professional associations and trade unions), the variables *workload intensity* and *work–home* *life balance* were added to reflect the unprecedented job demands of the COVID-19 pandemic and ongoing high demand for care.

- Reference criteria

Using an independent samples design, participants were randomly assigned to one of two reference frames: *“How important are the following issues to explain why* [respondent profession inserted] *staff leave the NHS”*or *“What needs to change to encourage* [respondent's profession inserted] *to continue working for the NHS?”*.

The rationale for asking current NHS employees about the behaviour of colleagues rather than themselves was twofold. First, asking respondents to rate reasons to leave is not salient to those who have no intention of leaving. Second, the benefits of accessing staff insights were judged to outweigh the risk of causal attribution error. Moreover, such an error can reasonably be assumed to be common, i.e. to cancel out, such that it is discountable between conditions (stay and leave frames) and job families, such that relative differences can be considered robust. Additionally, insight into employee beliefs, accurate or otherwise, is of value in signposting the need for employer intervention.

### Data collection

The data were gathered from July to December 2021 within the second wave of an online quantitative survey of influences on NHS staff retention. Ethical approval for the research was granted by the University of Bath Department of Psychology Ethics Committee (reference: PREC 20–259); a requirement for NHS governance approval. The Health Research Authority (HRA) did not require NHS Research Ethics Committee (REC) review; NHS Integrated Research Application System IRAS Project ID: 293153. Respondent participation was voluntary and anonymous. Informed consent to participate was obtained from all of the study participants.

### Recruitment and study sample

Participants were a UK-wide opportunity sample recruited via the YouGov panel, UNISON trade union members and 12 NHS Trusts in England, using electronic mailing and newsletter communications to distribute the survey link. A breakdown of the sample by health profession job-family for the *leave* and *stay* conditions is given in Table 1.

**Table 1:** Sample of health professionals relative to percentage employed within the NHS

Profession job-family	Numbers employed	Leave	Stay
Doctors (consultant and specialist inc.’ trainees)^a^	124,000 (15%)	227 (12%)	205 (11%)
Nursing professionals and midwives^b^	332,000 (40%)	687 (35%)	666 (36%)
Nursing non-professional^c^	280,000 (33%)	383 (20%)	320 (17%)
Allied health^d^	84,000 (10%)	417 (21%)	422 (22%)
Ambulance paramedics and technicians	18,000 (2%)	243 (12%)	258 (14%)
Total	837,000	1,957	1,871

**Note(s):** Relative proportions based on NHS England (only) figures at the time of data collection)

a,b,c NHS England Staff in post statistics June 2022. https://digital.nhs.uk/data-and-information/publications/statistical/nhs-workforce-statistics/june-2022. d Michas (2022)

The samples for each job-family are substantially larger than the minimum number considered sufficient to produce a reliable scaled output (Comer *et al.*, 1984). It should be noted that interval scales generated by the method paired comparisons are not subject to variability due to the sample size or influenced by variation between the proportions in the realised sample and actual proportions within the NHS workforce they represent (Ryan *et al.*, 2001).

### Procedure

Under both conditions, participants were asked to indicate the more important issue for each randomised pairing of variables; a total of 28 judgements per participant. Paired comparison is a forced choice method and there was no option to rate paired variables equally and no option to revisit or revise earlier judgements in the presented sequence. The task took five to eight minutes to complete (for a copy of the question set, see supplementary file).

### Analysis

#### Pre-analysis check of within-respondent consistency

Tests of within-respondent consistency (Kendall K) on a randomly selected sub-sample (*n* = 60), indicated that 94% of participants exhibited acceptable consistency (*K* = />0.70) in item ranking (A > B > C …), indicating that the item set could be considered suitable for scale development (Thurstone, 1927; Bock and Jones, 1968) and testing of contrasts.

## Results

### Comparison of leave and stay scales for all job families (pooled data)

**Figure 1:**
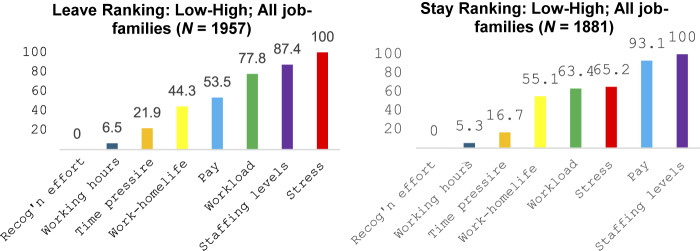
All-job-families (pooled data) – leave and stay scales. Source(s): Authors’ own work

The global (pooled data for all job families) scales for *leave* and *stay* were determined by transforming the judgement proportion (the frequency with which each variable within the respective data set was rated as more important than all of the other variables) to produce a 0–100 ratio scale. The lowest rated variable is set to 0 and the highest to 100 (Figure 1).

At the level of rank order, formal testing of the degree of alignment between the *leave* and *stay* frames indicated a strong relationship (Tau = 0.786, *p* = 0.008; *R*^2^ = 0.687). The degree of alignment presents as strongest for the three lowest ranked variables, with greater variability amongst the highest ranked variables, notably with respect to pay.

The degree of job-family alignment was strongest between doctors, allied health and nursing professionals, with paramedics presenting as an outlier. Job-family comparison findings (Tau and *R*^2^) are provided in Appendix Table A2.

### Comparison of leave and stay scales within job families

**Figure 2:**
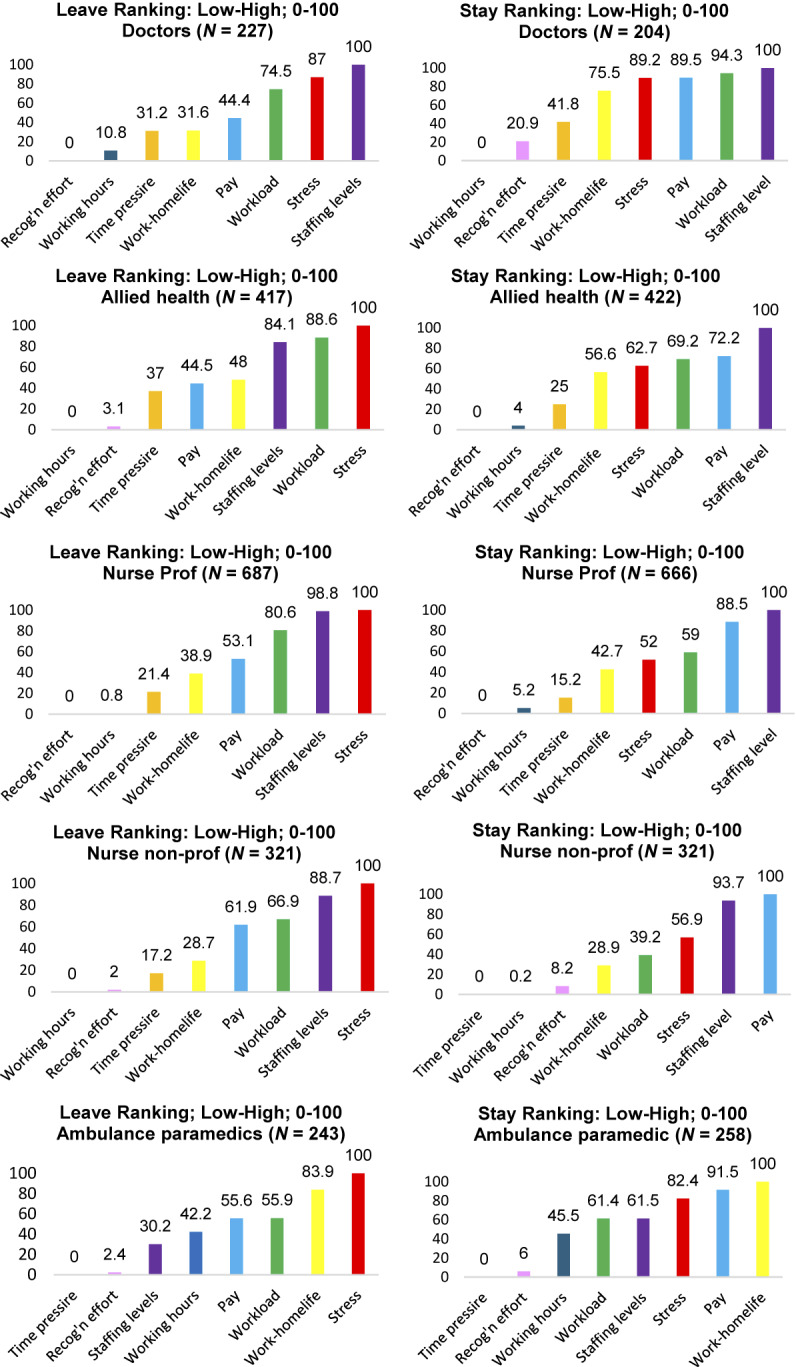
Within job-family – leave and stay scales. Source(s): Authors’ own work

Figure 2 highlights the primacy of sufficiency in staffing levels for both *leave* and *stay* frames as a consistent finding for each job-family, with the exception of paramedics, who rated it as sixth (*leave*) and fifth (*stay*) most important. Work–home life balance presents as more salient for paramedics than the other job families and was ranked second highest (*leave*) and highest (*stay*). Pay (all job families) was notably and consistently ranked more highly within the stay than the leave frame. It was ascribed a first/second position for four of the five job families within the *stay* frame. By contrast, mental health/stress, which was consistently rated highest or second highest in the *leave* frame, was mid-ranked in the *stay* frame. The degree of alignment between job families and stay/leave frames was greatest with respect to the ranking of working hours and recognition of contribution, with both appearing towards the bottom of the distribution.

The degree of alignment between *leave* and *stay* profiles within each job-family (Figure 2) was modest (range Tau = 0.500–0.643; see Appendix Table A3), which contrasted with the relatively strong relationship identified within the pooled sample.

### Exploring job-family contrasts in the ranking of other variables relative to pay and staffing

The output from paired comparisons is a subjective scale of relative differences on a continuum between variables within the item set; it is therefore desirable to anchor these to a benchmark that can be considered to possess a determinable objective value (Thurstone, 1959; Östberg, 1980). Pay and staffing level meet this criterion, accepting that respondents' judgements of the sufficiency of both pay and staffing level reflect subjective assessments rather than other stakeholder claims over absolute values/rates. Moreover, both are topical and the subject of much debate with respect to staff retention and the future of the NHS (Exworthy and Nash, 2024; Krachler and McGivern, 2024; Morris, 2022). The purpose here was to explore job-family contrasts in ratings of the other variables relative to pay and staffing level. This has implications for the impact of intervention activity limited to enhancement of pay and or staffing level. There is no suggestion that the importance of pay or staffing can be considered “equal” across the job families. The point of interest rests with job-family contrasts in the weighting of the other variables relative to the standardised items.

**Figure 3:**
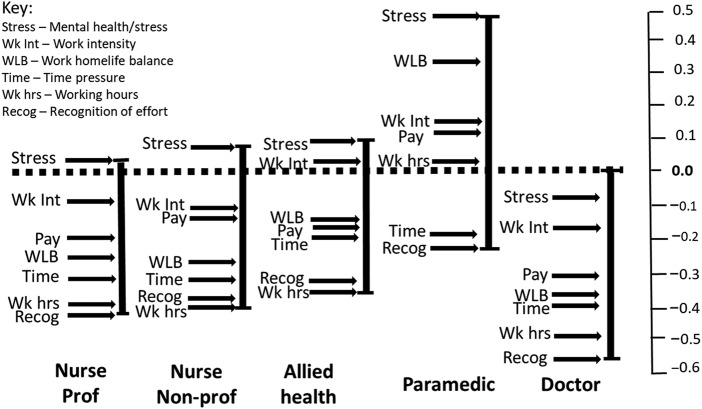
Job-family scales relative to rating of staffing – leave frame. Source(s): Authors’ own work

**Figure 4:**
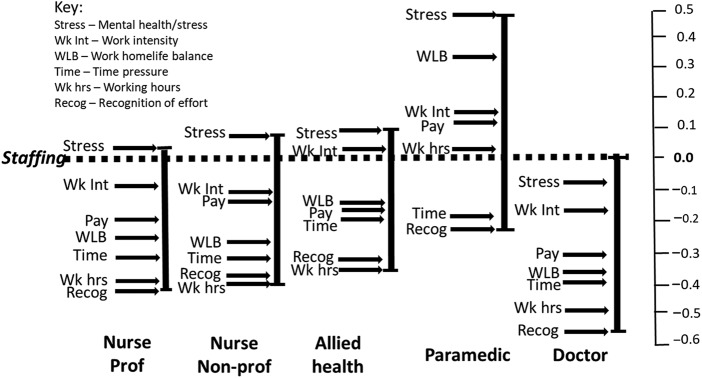
Job-family scales relative to rating of staffing – stay frame. Source(s): Authors’ own work

**Figure 5:**
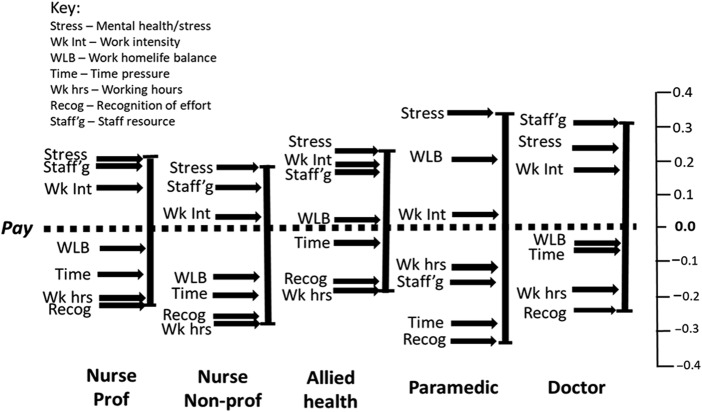
Job-family scales relative to rating of pay – leave frame. Source(s): Authors’ own work

**Figure 6:**
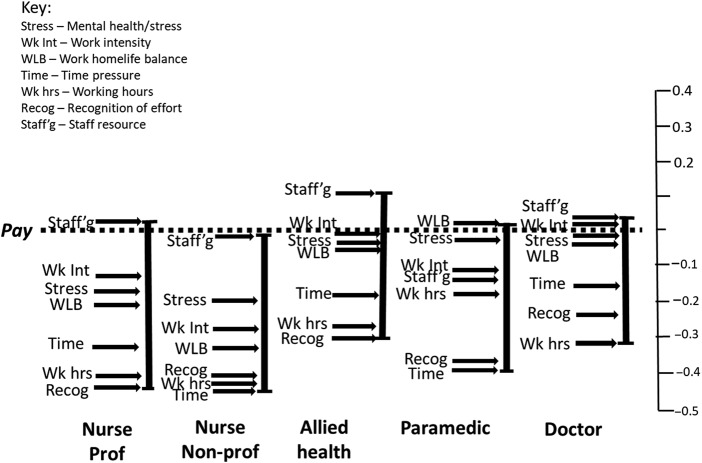
Job-family scales relative to rating of pay – stay frame. Source(s): Authors’ own work

The product of this was to scale the *leave* and *stay* output for each job-family relative to staffing level (Figures 3 and 4) and pay (Figures 5 and 6), i.e. the relative weightings of the other assessed variables relative to staffing level and pay.

### Formal testing of contrasts

Statistical testing explored contrasts between and within job families and the stay/leave frames relative to pay and staffing level. Reflecting recommendations on statistical testing of contrasts for paired comparisons output, the scales values were transformed to arcsine deviates to enhance their approximation to a normal distribution (Sjoberg, 1967).

#### Contrasts relative to pay

**Figure 7:**
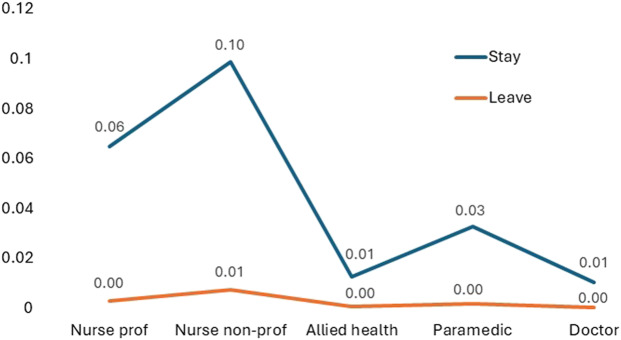
Leave and stay scale mean contrasts by job-family relative to pay. Source(s): Authors’ own work

Testing of differences (ANOVA) detected a main effect with respect to the salience of pay relative to the other variables F(1, 60) = 14.537, *p*=<0.001, which was confirmed to be higher within the *“what needs to change to motivate staff to stay’* than the *‘reasons why they leave”* (Figure 7 and Appendix Table A4). No other contrasts achieved significance at *p*= <0.05.

Univariate testing (independent samples *t*-test) of within-job-family contrasts between the stay and leave frames revealed no differences for doctors, allied health or paramedics, but a contrast was identified for nurses in their rating of pay as higher (more important) within the *stay* than the *leave* frame (nursing professionals *leave* M = −0.02, SD = 0.19; *stay* M = −0.11, SD = 0.15; *t*(12) = −2.36, *p = *0.018 and nursing non-professional *leave* M = −0.09, SD = 0.27; *stay* M = −0.31, SD = 0.16; *t*(12) = −2.40, *p = *0.034).

#### Contrasts relative to staffing level

**Figure 8:**
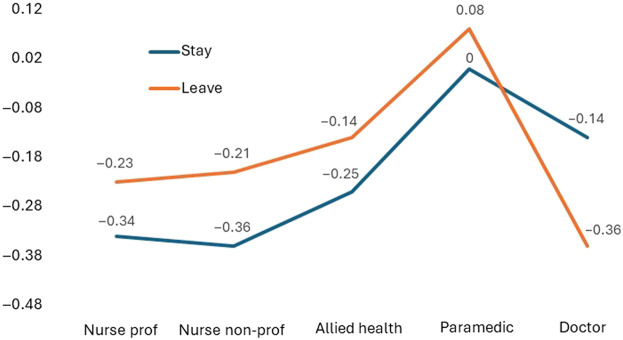
Leave and stay scale mean contrasts by job-family relative to staffing. Source(s): Authors’ own work

Testing of differences (ANOVA) detected a main effect with respect to staffing and job-family F(2, 60) = 6.488, *p*=<0.001. Post-hoc testing indicated that staffing level presents as less salient for paramedics in the array of *leave/stay* variables than the other job families; the degree of contrast presenting as greater in the leave than the stay frame (Figure 8 and Appendix Table A4).

Univariate testing of within job-family contrasts between the leave and stay frames relative to staffing level detected a more prominent profile for doctors in the *stay* frame (M = −0.15, SD = 0.14; *t*(12) = 2.45, *p = *0.030) than the *leave* frame (M = −0.02, SD = 0.18). Action to enhance staffing levels is presented as being of greater importance as a reason to stay for doctors. No equivalent contrasts between the *stay* and *leave* frames with regard to staffing level were detected for the other job families.

## Discussion

Staffing level, mental health/stress and workload intensity were the highest-ranked variables in both *leave* and stay frames. Improvement to staffing level exhibited consistent primacy across both frames and all job families, excepting paramedics. Paramedics present as an outlier to the general profile in terms of their respective weighting of variables, in particular the impact of improvements to staffing and pay. Pay is ranked higher as an issue that needs to change to motivate or enable staff to stay than as a reason why staff leave, with this contrast being greatest for nursing staff (professional and non-professional). Our central finding that the alternative frames produce different relative weightings for a common set of variables aligns with recent studies within and outside the health sector. It questions claims and assumptions that reasons to leave and reason to stay are mirror images (Coombs *et al.*, 2010; Cho *et al.*, 2009). This finding suggests that to produce a comprehensive perspective on priorities for intervention, employers need to consider both frames.

The most notable *leave* versus *stay* domain contrasts were in respect of pay and mental health/stress. Contrasting with a significant proportion of contemporary industrial relations claims (Gordon, 2022; Gregory, 2022; Morris, 2022), pay did not feature prominently as a reason to leave and was mid-ranked. However, it was ranked highest or second highest within the stay frame by allied health professionals, nursing staff (professional and non-professional) and paramedics. Similarly, the universal primacy ascribed to stress within the leave frame contrasted with its mid-ranked position within the stay frame. Pay represented the most marked between-frame contrast, being mid-ranked as a reason why staff leave but showing primacy in relation to the need for change. Its mid position as a reason to leave may reflect prevailing pay rates in job-opportunities outside the NHS, in particular, private sector healthcare. The available evidence indicates that, except for doctors, UK private sector healthcare pay rates are strongly aligned with NHS rates. There is also evidence that health professional migrations to non-health sector employment tend to result in lower pay (Weyman *et al.*, 2013; Quaile, 2016). Speculatively, the higher profile of pay within the stay frame may reflect a perceived effort-reward imbalance in the context of unprecedented rises in workload post-2020 that is fuelling perceptions of unfairness, inequity and injustice (Adams, 1965; Krachler and McGivern, 2024). This can be characterised as “*if I am being asked to continue working under these conditions I want more pay”*. However, it is also possible that the prominence of pay as a stay factor could be subject to cognitive bias. Availability bias can inflate the salience of simple intuitive solutions (Kahneman, 2011). Relatedly, attention to pay was the only future-facing variable (reason to stay) amenable to *immediate* change with tangible benefits for staff (Kahneman, 1979).

The above should not be interpreted as diminishing the importance of pay to NHS employees; there is ample evidence of significant dissatisfaction in both relative and absolute terms (Propper *et al.*, 2021; Charlesworth, 2024). Rather, our findings on pay show alignment with the substantial body of evidence that intervention to increase pay alone may have a modest impact in resolving the retention issue in the absence of attention to other key drivers, most prominently staffing level, work intensity and stress (Arnold *et al*., 2003; Arnold *et al.*, 2006; Joshua-Amadi, 2003; Shen *et al.*, 2004; Robinson *et al.*, 2005; Frijters *et al.*, 2007; Drennon *et al.*, 2016; Bimpong *et al.*, 2020; Exworthy and Nash, 2024).

Stress, while prominent in both frames, was ascribed greater salience for *leave* than *stay*. A plausible explanation for this contrast is that stress in the *leave* frame is perceived by staff as a consequence of other important variables such as staffing level and workload, essentially presenting as a trail indicator in relation to job demands and workload as reasons to leave. The greater salience of job-demands and workload as reasons to stay is consistent with this. If viewed from a stress risk mitigation perspective, the primary causes of stress within the stay frame are ascribed a higher profile and present as lead (precursor) indicators (Leveson, 2013). It also seems possible that the weighting of stress within the stay frame may be influenced by prior experience of employer practice and arising intuitions with respect to *what is on offer* to mitigate stress. Specifically, the contemporary emphasis on individual-level resilience training risks conveying the implicit message that staff must endure poor working conditions (Mehta *et al*., 2024). The more modest ranking of stress within the future-facing stay frame may reflect perceptions of *more of the same*, rather than interventions to address systemic root causes such as job-demands (Taylor, 2019).

Paralleling earlier findings (Weyman *et al*., 2020), the consistent low rank ascribed to working hours in both frames and across all job families raises a question over the prominence of changes to working hours, in particular, enhanced flexibility as a key solution to the retention challenge within contemporary NHS staff retention guidance (Dean, 2018; NHS Improvement, 2018). As with our conclusions on pay, this does not diminish the value of choice and flexibility over working hours to staff, but it does suggest that changes to working hours in the absence of attention to higher-ranked variables may have a modest impact.

The most marked difference between the job families was with respect to paramedics, who appear as something of an outlier. In particular, their lower ranking of staffing level and higher ranking of work-home-life balance. While the salience of staffing may be attenuated for paramedics due to working predominantly in pairs or alone rather than in teams, this interpretation seems less credible given the high profile (sector and national) media coverage of on-going staff shortages within the ambulance service and claims of it being higher than in other NHS functions (Wooler, 2023; Alarilla *et al.*, 2022; Hayes, 2022; Quaile, 2015). Treating staffing level as a constant that has equal relevance across the job families and a benchmark against which to scale other variables, sponsors a different conclusion in relation to paramedics. Staffing level is not unimportant to paramedics, the variables paramedics rank above this benchmark (stress, pay, working density and work–home life balance) are more important in their stay-leave decisions compared to other job families. The implication for employers is that while a case for intervention to address staff shortages shows primacy across all job families, intervention on this variable alone may have less impact on improving paramedic retention than the other segments of the labour force.

Ratings of work-home life balance and the relatively higher weighting of working hours by paramedics represented a contrast with the other job families. A number of sources report linkages between these variables amongst paramedics (Wooler, 2023; Weyman and O'Hara, 2019; Kirby *et al*., 2016). A notable feature of paramedic working hours is that their shift patterns tend to be significantly more irregular and less predictable than other job families (McCann, 2022; Weyman and O’Hara, 2019; Kirby *et al.*, 2016) with greater potential for disruption of home life. While the irregularity of shift patterns may reflect service providers’ need to manage fluctuations in the demand for care, the impact of institutional pressure to meet response time targets and unpredictable late finishes (Hayes, 2024; Barth *et al*., 2022) invites a more strategic approach to staff deployment (Chartered Institute of Personnel Directors, 2022; Eaton *et al*., 2022). The finding that a notably higher proportion of paramedics report applying for a non-NHS job (24%, *N* = 1157), compared with the all-staff rate of 13% (*N* = 9,220) in the six month period prior to completing the paired-comparisons, task suggests that paramedics represent a priority job-family for bespoke intervention.

Staffing level was also a source of variability for doctors. Here, the contrast was not with other job families, but between the leave and stay frames, whereby enhancement of staffing levels was ascribed greater salience as a reason to stay than was staff shortage as a reason to leave. Reasons for this cannot be determined on the basis of the ranking task evidence and published findings afford no directly relevant insight. However, the finding of differences suggests that the issue may merit further investigation.

The detection of differences in the profile of leave and stay frames suggests that the more commonly encountered research and employer focus on the former may only produce a partial perspective. This finding shows alignment with other research from within and beyond the healthcare sector that points to differences between leave and stay frames (Chami-Malaeb, 2018; Ghazali *et al.*, 2018; Lee *et al*., 2020; HakemZadeh *et al.*, 2024). In this respect, we would endorse Chami-Malaeb's conclusion that “The intention to leave and intention to stay may not be similar and cannot be considered to be the opposite of each other despite the fact that in most prior studies, the terms, desire to remain or intention to stay and intention to leave, have been used interchangeably.” p.79 (Chami-Malaeb, 2018).

World-wide health professional shortages, increased mobility of the healthcare workforce and perceived attractiveness of health systems in other countries are potential factors influencing stay/leave intentions. Evidence reviews and cross-national studies show variation in the extent of turnover/attrition in different countries. For example, Bruyneel *et al*. (2025) observed significant differences in work environment, perceived workload and intention to leave for nurses across six European countries. The authors attribute this finding to variations in national policies and practice (e.g. paid leave, work hours, management, workload, working environment). A review of why UK health professionals are leaving the NHS identified factors attracting staff to other countries, although mentioned less frequently than reasons to leave, included expectations of better pay, working conditions and work–life balance (Onyejesi *et al.*, 2025). Feeling overworked was the most frequently cited reason for leaving. These insights suggest there is merit in learning from the approaches of other countries to help enhance retention strategies and reasons to stay in the UK.

For the NHS, the findings indicate the primacy of a need for enhancement in the areas of staffing, workload intensity and pay. They also raise questions over the current intervention emphasis on lower ranked variables, notably working hours and recognition of effort (National Finance Adacemy, 2022; NHS Employers, 2023). This is not to suggest that these variables are not important, but raises questions over whether action to address variables at the lower end of the distribution will make enough of a difference in the absence of attention to higher ranked influences on employee stay and leave behaviour. The findings provide NHS workforce policy and planning functions with a point of comparison for existing staff retention intervention priorities and practices. However, synthesis is required to map the boundaries of the scope for intervention to address the higher-ranked variables, as the identification of priorities for intervention involves more than simply selecting variables from the top of the *leave* and *stay* rankings. There is a need to take account of the scope for influence and impact, the configuration of which will vary by topic, job-family and timeframe (short versus long-term).

Arising implications for NHS policy makers and employers are: (1) Action to address staff shortages shows primacy in both frames, and for most staff in most roles. (2) A comprehensive perspective on staff retention requires consideration of the *leave* and *stay* effect of headline variables. (3) Evidence of job-family differences in the weighting of *leave*-*stay* variables suggests that a segmented approach is likely to be more effective in delivering bespoke interventions and higher rates of return on intervention investment than a whole population (“one size fits all)” approach (Karanika-Murray and Weyman, 2013).

## Conclusions

The alternative frames of: *Why do staff who do your type of job leave* *NHS* *employment?* or *What needs to change to make staff who do your type of job stay in* *NHS* *employment?* Produce a number of contrasts in the ranking and weighting of widely cited influences on staff retention across the principal care-provider job families. Staffing level was the most widely and consistently reported priority for improvement. However, gains from intervention on this issue alone may be proportionally less for paramedics. Improvement in pay shows prominence within the stay domain but is outranked by job-demands and workload as a reason why staff leave.

The detection of domain differences suggests that it is desirable for researchers, policy makers and employers to gather data on both *leave* and *stay* influences to produce a comprehensive perspective. Similarly, the detection of job-family contrasts suggests that there may be gains from bespoke intervention packages for each. Paramedics present as an outlier that would benefit from tailored intervention, notably with respect to their higher weighting of work–home life balance and working hours, but also the finding that relative to staffing level, work–life balance, pay, stress and work intensity are bigger influences on retention than in other job families. The relationship and basis of differences between reasons to leave and reasons to stay remains under-researched and under-theorised. An action theory perspective might, for example, prove illuminating.

These insights are of relevance to future NHS human-resource strategy in signposting priority variables and job families for intervention. The capacity to identify and monitor the variables that have the greatest impact on staff retention potentially affords the biggest returns on intervention investment. Our study demonstrates contrasts between the principal secondary care job families but this does not represent the limit of demographic contrasts that may be relevant to explore, for example ethnicity, staff grade, tenure, age and gender, as well as administrative, ancillary and other support functions.

While there is evidence of increasing adoption of initiatives designed to enhance historically low exit interview rates and broaden the scope of data capture (Van den Oetelaar *et al*., 2021), the focus appears to remain on reasons for leaving. From a risk management perspective trail data affords limited insight to identify incubating issues before they become critical or feedback on the effectiveness of intervention activity aimed at increasing retention. Regular (e.g. annual) monitoring of *leave*-*stay* variables as addressed in this study, could provide more informative data on precursor variables for risk management purposes (Dean, 2018) and has the potential to provide feedback on the impact of any changes. The NHS and its constituent employers would benefit from the development of a staff survey “tool” focused on lead indicator variables to benchmark and track the staff retention climate and provide early-warning of incubating issues. Moreover, the breadth of relevant precursor variables impacting on staff health, well-being and commitment to remain could profitably be expanded beyond those explored within the study reported here. Similarly, the perspective on segmentation of the workforce could be explored at a greater level of granularity with a view to enhancing the fit between intervention activity and the needs of different demographics.

### Strengths and limitations

The analysis reported here is based upon what is believed to be the first large-scale systematic attempt to determine the relative weighting of widely cited influences on NHS staff retention as variables impacting on staff orientation to leave or stay, and the extent to which profiles vary between the principal secondary care job families. The output is relevant to determining human resource priorities regarding salient staff issues and job families, to inform intervention aimed at stabilising and increasing staff retention rates. The capacity to determine this embodies the potential for enhancing the impact of human resources intervention relative to investment and reflects the tenets of evidence-based practice and organisational learning. With respect to perspective, the study contrasts with the greater (by volume) proportion of studies examining staff retention that focus on reasons why staff leave. It shows alignment with and adds to the evidence base that questions the implicit assumption of leave focused studies, that the weighting of *stay* influences is a mirror image of the leave profile (Lee *et al*., 2020; HakemZadeh *et al.*, 2024).

Methodologically, paired comparisons has been empirically demonstrated to offer a number of advantages over commonly used preference elicitation methods, notably direct ranking and Likert scales in relation to reproducibility (Thurstone, 1959; Bock and Jones, 1968; Comer *et al.*, 1984). Additionally, the interval scaled output points to the relative salience of the variables in the set. Variables can also be weighted to a respondent's subjective estimates of known quantifiable benchmarks, in the current instance pay and staffing level.

The decision to ask participants about their perceptions of peers, rather than their own stay or leave intentions, was essentially a product of sampling logistics and the significant barriers to accessing a large sample of employees who have left NHS employment. While it would be unreasonable to assume that perceptions of others will be free from attribution error, there are no known grounds for assuming that this would not be common across the different job families and leave versus stay samples. Thus there are grounds for regarding it as a source of common error that *cancels* *out*, such that detected relative contrasts can be considered reliable. Respondent ratings were informed by observation of the experiences of colleagues and not just themselves, which might be considered a strength. However, it remains possible that their intuitions, sense-making and attributions could be inaccurate.

There may also be grounds for considering a degree of imbalance to be present in the reliability and predictive strength of leave compared with stay frames. While responses to the questions *Why do staff who do your type of job leave* *NHS* *employment?* and *What needs to change to make staff who do your type of job stay in* *NHS* *employment?* are essentially impressionistic and subjective, the former is potentially more grounded in experience, and the latter more speculative. Although reference to both frames affords the more comprehensive insight, the relationship to staff behaviour may be stronger for the leave frame.

The *leave* and *stay* frame samples are substantial, concurrent, of comparable size and distribution and with participants randomly assigned to each. The job-family sub-samples showed strong alignment with contemporary NHS staff in post ratios for doctors, allied health professionals, nursing professionals (including midwives), but under-represent nurse non-professionals (support staff) and over-represent ambulance staff. However, the smallest sub-sample exceeded accepted norms regarding the minimum number of participants for scale development by a factor of 10 (Rajae-Joordens and Engel, 2005). Also, it is important to note that the output (scales) from the method of paired comparison is not subject to variability due to sample size or influenced by variation between the proportions in the realised sample or actual proportions within the NHS workforce. However, it is acknowledged that, in common with other voluntary surveys, the potential for self-selection response bias cannot be discounted.

## Supplementary Material

Data supplement

## Data Availability

The data relating to this study is stored in a secure repository at the university of Bath and is available on request from the corresponding author.

## Appendix

**Table A1:** Summary of variables identified as impacting on NHS health professional retention – for variable set

Fleming and Taylor (2007)	Smith and Secombe (1998)	Arnold *et al*. (2003)	Shen *et al*. (2004)	Joshua-Amadi (2003)
Community care	Nurses	Nurses and allied health	Midwives and consultants	Nurses
Working hours	Inflexible working hours	Working hours	Working hours/patterns	
	Inadequate resources	Under-staffing		
Pay	Inadequate pay	Pay		Low inequitable pay
Qualifications and training	Inadequate personal development opportunities			
Workload pressures	Excessive workload		Workload	Increased workload
Supervision and support				Personal isolation
	Inadequate promotion prospects/career structure			
			Bureaucracy and lack of autonomy	
			Job-stress	
			Lack of recognition	Lack of appreciation
Job satisfaction				
Client attitudes				
				Decline in patient care

**Source(s):** Authors’ own work

**Table A2:** Comparison of job-family *leave* and *stay* rankings (Tau) and relative ratings (*R*^2^)

Job-family	Leave		Stay	
	Tau	*R* ^2^	Tau	*R* ^2^
Doctors v Nursing prof	0.923	0.970	0.857	0.793
Doctors v Nurse non-prof	0.857	0.903	0.640	0.651
Doctors v Allied health	0.714	0.934	0.857	0.913
Doctors v Paramedic	0.357	0.573	0.214	0.433
Nursing prof v Nurse non-prof	0.923	0.970	0.764	0.912
Nursing prof v Allied health	0.857	0.976	1.0	0.929
Nursing prof v Paramedic	0.428	0.690	0.357	0.429
Nurse non-prof v Allied health	0.857	0.948	0.857	0.912
Nurse non-prof v Paramedic	0.375	0.707	0.571	0.401
Allied health v Paramedic	0.500	0.724	0.357	0.447

**Source(s):** Authors’ own work

**Table A3:** Within job-family relationship (Tau) between leave and stay rankings and relative ratings (*R*^2^)

Job-family	Tau	*R* ^2^
Doctors	0.643	0.696
Allied health	0.571	0.720
Ambulance paramedic	0.500	0.650
Nursing professional	0.643	0.572
Nurse non-professional	0.500	0.336

**Source(s):** Authors’ own work

**Table A4:** Contrasts relative to staffing level and pay

	Leave	Stay	
	Mean (SD)	Mean (SD)	*P = *
*Relative to staffing level*			
Doctors	−0.355 (0.183)	−0.145 (0.133)	0.030
Allied health	−0.160 (0.162)	−0.253 (0.132)	
Paramedics	0.090 (0.266)	−0.023 (0.174)	
Nurses professional	−0.244(0.165)	−0.330(0.167)	
Nurses non-professional	−0.224(0.178)	−0.364(0.331)	
*Relative to pay*			
Doctors	0.020(0.226)	−0.102(0.144)	
Allied health	0.033 (0.173)	−0.113(0.158)	
Paramedics	−0.043(0.267)	−0.187 0.161)	
Nurses professional	−0.185(0.189)	−0.265(0.185)	0.018
Nurses non-professional	−0.086(0.196)	0.314(0.157)	0.034

**Source(s):** Authors’ own work

**List of abbreviations**
COVID-19: Coronavirus disease 2019, alternatively termed: 2019 novel coronavirus and 2019-nCoV
NHS: National Health Service
F: F-ratio, the ratio of the between group variance to the within-group variance
P: Statistical probability
SD: Standard deviation
UK: United Kingdom

## Notes

The supplementary material for this article can be found online.
