# Supplementary material for: Framing effects in factors influencing health professional staff decisions to leave or stay working in the UK National Health Service
Source: J Health Organ Manag. 2026 Feb 20;40(9):131–52. doi: 10.1108/JHOM-10-2025-0638 (PMC13012393; doi:10.1108/JHOM-10-2025-0638)
Supplement: Data supplement [file jhom-10-2025-0638_suppl1.docx]

**A2.** Survey question set - leave and stay paired ranking task

**Question Text:** Either paragraph A or B depending on randomised allocation of participants

A: We would like your views in more detail on how important the following issues are to explain why staff who do your type of work *leave the NHS*. To make the task easier you will be presented with the issues two at a time. From each pairing, choose the issue that you think is the more important reason for people in your [profession inserted] leaving the NHS.

B: We would like your views in more detail on what needs to *change to encourage* [respondent’s profession inserted] *to continue working for the NHS?* To make the task easier you will be presented with the issues two at a time. From each pairing, choose the issue that you think is the bigger priority for change to encourage and support staff to continue working in the NHS.

**Presentation of items <1-8>:** The order of item pairs is randomized for each participant. Presentation of pairs includes the final line of text A or B to remind respondents of the framing.

| **[Q1]** | <1> | Time pressure |
| --- | --- | --- |
|  | <2> | Staffing levels |
| **[Q2]** | <1> | Time pressure |
|  | <3> | Working hours |
| **[Q3]** | <1> | Time pressure |
|  | <4> | Workload (intensity of work) |
| **[Q4]** | <1> | Time pressure |
|  | <5> | Work/ home-life balance |
| **[Q5]** | <1> | Time pressure |
|  | <6> | Pay |
| **[Q6]** | <1> | Time pressure |
|  | <7> | Mental health/ stress |
| **[Q7]** | <1> | Time pressure |
|  | <7> | Mental health/ stress |
| **[Q8]** | <2> | Staffing levels |
|  | <3> | Working hours |
| **[Q9]** | <2> | Staffing levels |
|  | <4> | Workload (intensity of work) |
| **[Q10]** | <2> | Staffing levels |
|  | <5> | Work/ home-life balance |
| **[Q11]** | <2> | Staffing levels |
|  | <6> | Pay |
| **[Q12]** | <2> | Staffing levels |
|  | <7> | Mental health/ stress |
| **[Q13]** | <2> | Staffing levels |
|  | <8> | Recognition of contribution |
| **[Q14]** | <3> | Working hours |
|  | <4> | Workload (intensity of work) |
| **[Q15]** | <3> | Working hours |
|  | <5> | Work/ home-life balance |
| **[Q16]** | <3> | Working hours |
|  | <6> | Pay |
| **[Q17]** | <3> | Working hours |
|  | <7> | Mental health/ stress |
| **[Q18]** | <3> | Working hours |
|  | <8> | Recognition of contribution |
| **[Q19]** | <4> | Workload (intensity of work) |
|  | <5> | Work/ home-life balance |
| **[Q20]** | <4> | Workload (intensity of work) |
|  | <6> | Pay |
| **[Q21]** | <4> | Workload (intensity of work) |
|  | <7> | Mental health/ stress |
| **[Q22]** | <4> | Workload (intensity of work) |
|  | <8> | Recognition of contribution |
| **[Q23]** | <5> | Work/ home-life balance |
|  | <6> | Pay |
| **[Q24]** | <5> | Work/ home-life balance |
|  | <7> | Mental health/ stress |
| **[Q25]** | <5> | Work/ home-life balance |
|  | <8> | Recognition of contribution |
| **[Q26]** | <6> | Pay |
|  | <7> | Mental health/ stress |
| **[Q27]** | <6> | Pay |
|  | <8> | Recognition of contribution |
| **[Q28]** | <7> | Mental health/ stress |
|  | <8> | Recognition of contribution |

**Source(s):** Authors’ own work
